# Development and validation of a prediction nomogram for non-suicidal self-injury in female patients with mood disorder

**DOI:** 10.3389/fpsyt.2023.1130335

**Published:** 2023-04-17

**Authors:** Sixiang Liang, Dan Li, Xinyu Liu, Isadora Jiang, Jinhe Zhang, Jun Liu, Sha Sha

**Affiliations:** National Clinical Research Center for Mental Disorders and Beijing Key Laboratory of Mental Disorders, Beijing Anding Hospital, Advanced Innovation Center for Human Brain Protection, Capital Medical University, Beijing, China

**Keywords:** prediction model, mood disorder, non-suicidal self-injury, nomogram, LASSO regression

## Abstract

**Background:**

Non-suicidal self-injury (NSSI) is a highly prevalent behavioral problem among people with mental disorders that can result in numerous adverse outcomes. The present study aimed to systematically analyze the risk factors associated with NSSI to investigate a predictive model for female patients with mood disorders.

**Methods:**

A cross-sectional survey among 396 female patients was analyzed. All participants met the mood disorder diagnostic groups (F30–F39) based on the Diseases and Related Health Problems 10th Revision (ICD-10). The Chi-Squared Test, *t*-test, and the Wilcoxon Rank-Sum Test were used to assess the differences of demographic information and clinical characteristics between the two groups. Logistic LASSO Regression Analyses was then used to identify the risk factors of NSSI. A nomogram was further used to construct a prediction model.

**Results:**

After LASSO regression selection, 6 variables remained significant predictors of NSSI. Psychotic symptom at first-episode (β = 0.59) and social dysfunction (β = 1.06) increased the risk of NSSI. Meanwhile, stable marital status (β = −0.48), later age of onset (β = −0.01), no depression at onset (β = −1.13), and timely hospitalizations (β = −0.10) can decrease the risk of NSSI. The C-index of the nomogram was 0.73 in the internal bootstrap validation sets, indicated that the nomogram had a good consistency.

**Conclusion:**

Our findings suggest that the demographic information and clinical characteristics of NSSI can be used in a nomogram to predict the risk of NSSI in Chinese female patients with mood disorders.

## 1. Introduction

Non-suicidal self-injury (NSSI) refers to the intentional destruction of one’s body tissue without suicidal intent, and not for socially sanctioned purposes (1), including cutting, burning, scratching, banging, hitting, biting, punching, sticking sharp objects, and carving (2). Meanwhile, female were more likely to report using cutting, biting, scratching, pinching, hair pulling and interfering with wound healing than male (3). It is a major public health problem among patients worldwide. While the onset of NSSI peaks in adolescence around age 14–15 (4), it is also a highly prevalent behavioral problem among people with mental disorders. A variety of studies have documented the strong association between NSSI and the presence of a mental disorder, including unipolar and bipolar depression, anxiety disorder, substance use, cluster B personality disorder, and eating disorder (5–7).

Non-suicidal self-injury is a maladaptive behavior that can result in numerous adverse outcomes that they may constitute a peril to the subject’s physical integrity and increase risk of suicidal behavior (8, 9). In addition to being dangerous, NSSI may be a risk factor for future suicidal behaviors (10–13). Sometimes, NSSI is grouped with self-harm because it is often difficult to determine whether a patient has suicidal ideation clinically. But irrespective of the presence or absence of suicidal ideation, the act of self-harm is a very important risk factor for future suicide (14, 15). Therefore, it is important to identify risk factors for NSSI and establish strategies for early prevention.

As compared with the general population, people with mood disorders have limited ability to regulate emotions and cope with life events, which may increase vulnerability to self-harm (16, 17). Therefore, NSSI prevention in patients with mood disorders seems more necessary. Previous research has found that people with mood disorders are more likely to self-harm than those with other medical conditions (18), and studies showed that the incidence of NSSI in patients with bipolar disorder and unipolar depressive disorder was 52 and 37%, respectively (19). Meanwhile, NSSI was a strong prognostic variable for depressive symptoms, global functioning, emotional dysregulation, neuroticism in patients with mood disorders (19), and had twice the risk of suicide attempts than those of mood disorder patients without NSSI (20).

Previous clinical observations and cohort studies have reported some risk factors of NSSI, such as depressive symptoms, the female sex, chronic interpersonal stress, early-life adversity, impulsivity, long duration of illness, prior history of NSSI, rumination symptoms, comorbid alcohol use disorder, insomnia, and digital media use (21–26). Personality traits may also constitute a risk factor for NSSI, as articles have shown that that higher self-awareness and lower extroversion are both predictors of suicide (27). However, a prediction model based on demographic information and clinical characteristics, rather than individual analyses, has yet to be developed.

Many studies have shown that females are more prone to NSSI than males and that there are differences in their methods of NSSI. Furthermore, research indicated that women are 1.5–3 times more likely than men to report NSSI (3, 28–30). In comparison to females, males with NSSI showed less evidence of impairment and negative associations, including fewer symptoms of borderline personality disorder (BPD), lower levels of suicide and psychopathology, better quality of life, and lower rates of emotional and sexual abuse (31). In order to make a more specific prediction of NSSI, this study was only conducted in female patients.

The present study aimed to systematically analyze the risk factors associated with NSSI to investigate a predictive model for patients with mood disorders, specifically using the least absolute shrinkage and selection operator (LASSO) regression and nomogram.

## 2. Materials and methods

### 2.1. Participants

This study is a cross-sectional retrospective analysis using data extracted from inpatient medical records of Beijing Anding Hospital from 2019 to 2021. All participants were diagnosed by at least two attending psychiatrists independently and met the mood disorder diagnostic groups (F30–F39) based on the Diseases and Related Health Problems 10th Revision (ICD-10) (32). The participants’ personal information was deleted to protect privacy. All patients were previously informed and agreed that the information in their medical records could be shared anonymously for research purposes. The study protocol was approved by the Ethics Committee of Beijing Anding Hospital.

A total of 396 hospitalized female patients with mood disorders were included in this study. The researchers reviewed the patients’ complete medical histories and excluded patients with other psychiatric disorders, including schizophrenia, schizoaffective disorder, personality disorders, intellectual disabilities, and alcohol or drug abuse. Medication use, including mood-stabilizer, antipsychotic, antidepressant, or anxiolytic, did not affect the participants’ inclusion in the study.

### 2.2. Materials

The sociodemographic information and clinical characteristics were collected, including age, education level, duration of illness, age of onset, duration of first onset, medication time, number of hospitalizations, BMI, marital status, occupation, family history, social dysfunction, income, residential address, polarity at onset, psychotic symptom(s) at first-episode, and somatic disease.

Non-suicidal self-injury was defined as any form of self-harm without suicidal intent at any time in the past (33). The assessments of NSSI were completed by psychiatrists and were part of the medical records. The psychiatrists used clinical interview to determine that the patient did not have suicidal ideation at the time of self-harm. Based on the medical records, the patients were then classified into two groups: patients with NSSI (NSSI) and patients without NSSI (NSSI-N).

Social functioning refers to an individual’s ability to participate in organized or informal group activities (including family, friends, and peers) and public activities (34). As social individuals, humans tend to place a high value on everyday social interactions, and if these interactions are limited, life may be negatively affected (35). Social dysfunction is defined as an individual’s decreased ability to interact with family, friends, and peer groups and to participate in activities at previously established levels (34). Social dysfunction was assessed by the Personal and Social Performance (PSP) Scale (36), which includes socially useful activities (e.g., work and study), personal and social relationships, self-care, and disturbing and aggressive behavior (36). Patients were divided into two groups – with or without social dysfunction – based on a 70-point cut-off.

### 2.3. Statistical analyses

Continuous and categorical variables are described using mean (standard deviation) and counts (percentage), respectively. The chi-squared test and Wilcoxon rank-sum test were used to test the differences between NSSI and NSSI-N, and were based on baseline demographic and clinical characteristics. We used multiple imputation, based on five replications and a chained equation approach method in the RMI procedure, to account for missing data on income, marital status, BMI, number of hospitalizations, and occupation.

Logistic LASSO regression analyses were then used to identify the risk factors of NSSI associated with the demographic information and clinical characteristics. The logistic LASSO model is a shrinkage method based on regression methodology that can automatically eliminate the uninfluential variable from a large and potentially multicollinear set of variables. It helps produce a more relevant and interpretable set of predictors, and avoids overfitting (37). We used 10-fold cross-validation to choose the penalty term λ and computed the binomial deviation of the test data as a measure of the predictive performance of the fitted model. The built-in function in R produces two automatic λ: the left λ refers to the one that obtains the smallest binomial deviance among all λ values, and the right λ refers to the one that obtains the simplest model within a variance range. The standard errors of the LASSO coefficients were obtained *via* bootstrapping within the primary sampling unit and strata.

Using the risk factors screened out by the LASSO regression, we created a prediction nomogram by assigning a graphic preliminary score to each of the predictors with a point ranging from 0 to 100. The scores for each risk factor were then summed to obtain a total score, which was ultimately converted to the probability of a patient developing NSSI (from 0 to 100%).

The performance of the nomogram was evaluated by Harrell’s concordance index (C-index) and the calibration plot (38). Generally, C-index > 0.7 reflects a well-fitted feature of the predictive model. Independent significant variables were used to develop the nomogram. The internal validation was performed using the bootstrap method. In order to explore the accuracy of the prediction model, the researchers created the basic model using the following factors: age, marital status, occupation, education, family history, income, duration of illness, duration of first onset, somatic disease, and BMI.

The accuracy of the predictive model was tested using both calibration and discrimination viewpoints by adding significant risk factors. Integrated Discrimination Improvement (IDI) was used to evaluate the discrimination capability of significant risk factors (39, 40). Calibration capability was calculated using the -2log likelihood ratio test. Akaike Information Criteria (AIC) was used to assess the improvement in model fit after adding risk factors to the basic model (41). Furthermore, Decision Curve Analysis (DCA) (42) and Receiver Operating Characteristic (ROC) curves were also calculated in this study to evaluate the predictive ability of the model.

Statistical analyses were conducted using the STATA software special Release 14.0 (StataCorp, College Station, TX, USA). The LASSO regression, nomogram, DCA, and ROC curves were generated using R-language (version 3.5.2). The LASSO logistic regression model was generated with the “glmnet” package. The nomogram and calibration curve were generated with the “rms” package. The DCA was performed with the “dca.R,” and the ROC curves were plotted with the “pROC” package. *P* < 0.05 was considered statistically significant.

## 3. Results

### 3.1. Demographic characteristics

A total of 396 hospitalized female patients were involved in the present analysis (mean age: 35.81 years old, SD = 15.65), and 35.6% of the patients had NSSI. The patients’ age, duration of illness, age of onset, duration of first onset, number of episodes, marital status, occupation, polarity at onset, and psychotic symptom at first-episode were significantly different between the NSSI group and the NSSI-N group. Meanwhile, there were no differences in education level, medication time, BMI, family history, income, residential address, and somatic disease between the two groups (Table 1).

**TABLE 1:** Demographic information and clinical characteristics.

	NSSI (*n* = 141)	NSSI-N (*n* = 255)	*P*	χ^2^/z
Age (years)*	31.13 (16.22)	38.39 (14.73)	<0.001	5.03
Educational years (years)	13.40 (2.55)	13.33 (2.89)	0.543	−0.61
Duration of illness (months)	76.83 (96.89)	112.07 (112.82)	<0.001	3.77
Age of onset (years)	23.90 (13.51)	28.88 (13.25)	<0.001	4.78
Duration of first onset (months)	3.37 (5.0)	4.05 (4.86)	0.015	2.44
medication time (months)	6.32 (12.03)	6.97 (12.03)	0.056	1.91
Number of hospitalizations	1.77 (2.04)	2.10 (1.95)	0.009	2.62
BMI (kg/m^2^)	23.61 (5.38)	24.64 (5.96)	0.141	1.47
Marital status (n, %)			< 0.001	22.86
Unmarried	104 (73.8%)	125 (49%)		
Divorce/widowhood	8 (5.7%)	25 (9.8%)		
Married	29 (20.6%)	105 (41.2%)		
Job (n, %)			0.015	8.46
Manual labor	4 (2.8%)	14 (5.5%)		
Mental labor	63 (44.7%)	78 (30.6%)		
Unemployed	74 (52.5%)	163 (63.9%)		
Family history (n, %)	42 (29.8%)	74 (29%)	0.872	0.03
Social dysfunction	99 (70.2%)	121 (47.5%)	< 0.001	19.05
Income (n, %)			0.302	3.65
< ¥1000	27 (19.1%)	32 (12.5%)		
¥1000–3000	36 (25.5%)	72 (28.2%)		
¥3000–5000	38 (27%)	66 (25.9%)		
> ¥5000	40 (28.4%)	85 (33.3%)		
Residence (n, %)			0.755	0.56
City	108 (76.6%)	189 (74.1%)		
Villages and towns	8 (5.7%)	13 (5.1%)		
Countryside	25 (17.7%)	52 (20.8%)		
Polarity at onset (n, %)			< 0.001	16.11
Depression	129 (91.5%)	191 (74.9%)		
Mania	9 (6.4%)	48 (18.8%)		
Delusion	3 (2.1%)	16 (6.3%)		
Psychotic symptom at first-episode (n, %)	54 (38.3%)	71 (27.8%)	0.032	4.59
Somatic disease (n, %)	136 (96.5%)	244 (95.7%)	0.710	0.14

*Mean (SD); NSSI, patients with non-suicidal self-injury; NSSI-N, patients without non-suicidal self-injury.

### 3.2. Predictors selection and development of an individualized prediction model

**FIGURE 1:**
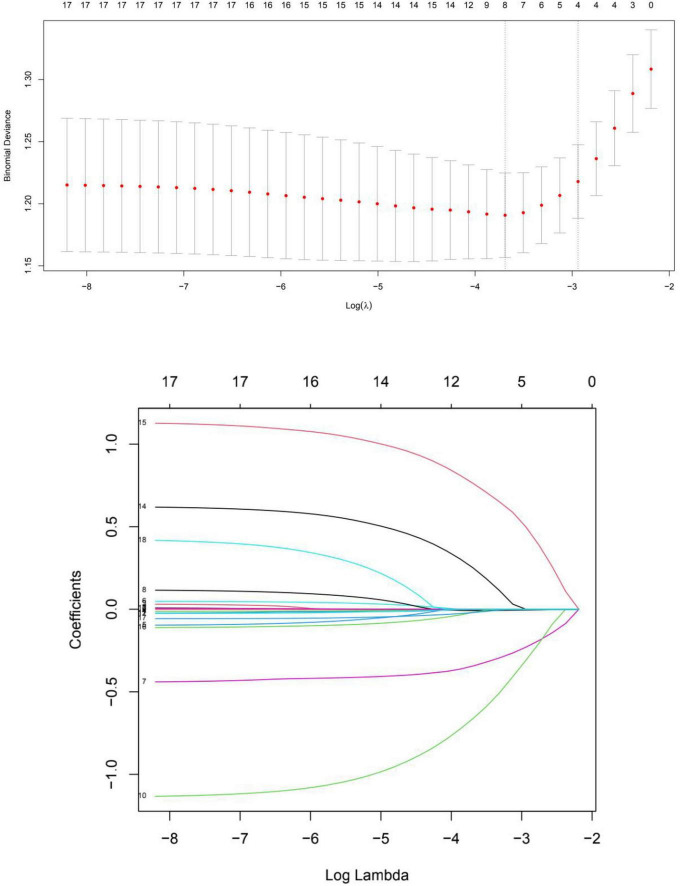
Cross validation plot for the penalty term and plots for LASSO regression over different values of the penalty parameter.

The 18 variables were included in the LASSO regression. We chose the left λ as it had the smallest binomial deviance, and after the LASSO regression selection (Figure 1), 6 variables remained significant predictors of NSSI: marital status, age of onset, polarity at onset, psychotic symptom at first-episode, social dysfunction, and number of hospitalizations.

The estimated coefficients for the LASSO regression are shown in Table 2, psychotic symptom at first-episode (β = 0.59) and social dysfunction (β = 1.06) increased the risk of NSSI, marital status (β = −0.48), age of onset (β = −0.01), polarity at onset (β = −1.13), and number of hospitalizations (β = −0.10) decreased the risk of NSSI.

**TABLE 2:** The estimated coefficients for LASSO regression.

Variables	Coefficients	Std. error
Marital status	−0.48	0.15
Age of onset	−0.01	0.01
Polarity at onset	−1.13	0.30
Psychotic symptom at first-episode	0.59	0.26
Social dysfunction	1.06	0.25
Number of hospitalizations	−0.10	0.06

### 3.3. Prediction model

**FIGURE 2:**
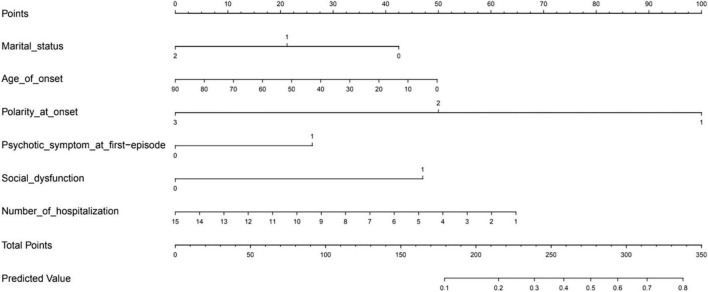
The prediction nomogram of risk factors for non-suicidal self-injury (NSSI) in patients with mood disorders.

As shown in Figure 2, a nomogram model was developed to predict the risk of NSSI based on the previous significant factors in the LASSO regression analyses, including marital status, age of onset, polarity at onset, psychotic symptom at first-episode, social dysfunction, and number of hospitalizations.

In the prediction model, in terms of marital status, unmarried was defined as 0, divorce or widowhood was defined as 1, and married was defined as 2. For polarity at onset, patients with depression as the primary form of onset was defined as 1, mania as the primary form of onset was defined as 2, and delusion as the primary form of onset was defined as 3. Lastly, patients without social dysfunction or psychotic symptoms at first-episode was defined as 0, otherwise 1.

**FIGURE 3:**
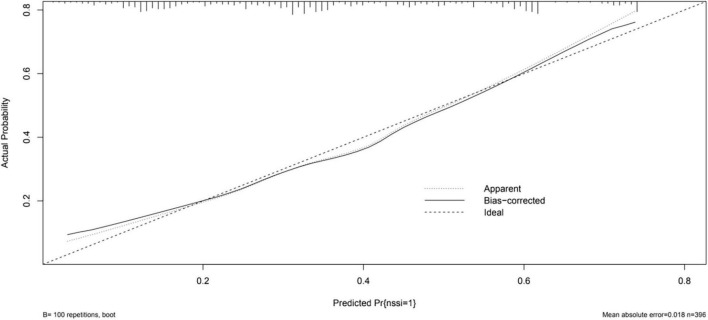
The logistic calibration curve of the prediction nomograms of risk factors for NSSI in female patients with mood disorders.

The calibration curve of the nomogram demonstrated good agreement between the predicted and observed risk of NSSI. The C-index of the nomogram was 0.74875, and turned into 0.73140 in the internal bootstrap validation sets (Figure 3). A c-index value of 0.70 or higher indicates that the nomogram had a good consistency.

### 3.4. Prediction accuracy assessment

**FIGURE 4:**
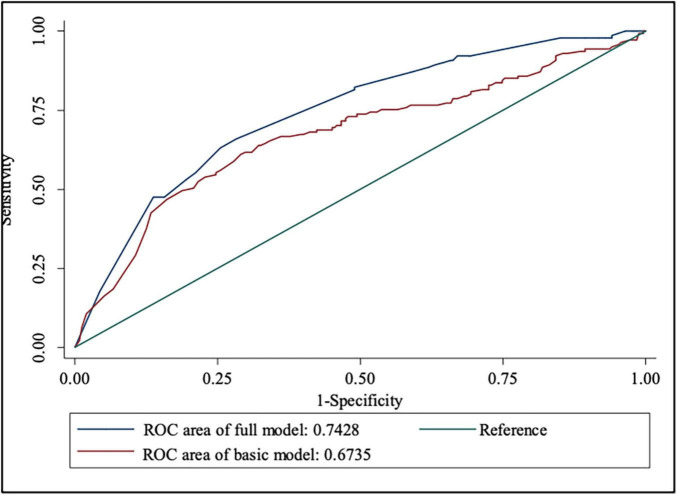
ROC curve for the prediction nomograms of risk factors for NSSI.

**FIGURE 5:**
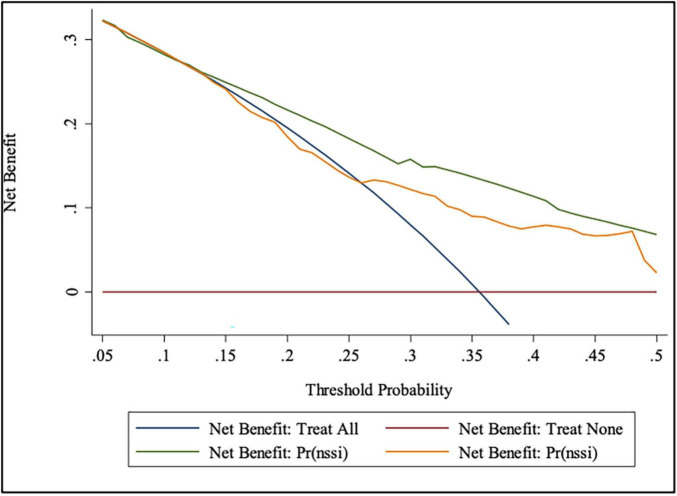
DCA for the prediction nomograms of risk factors for NSSI.

Table 3 shows the prediction accuracy obtained by separately adding aforementioned risk factors to the basic model. Akaike information criterion (AIC) is a criterion for measuring the goodness of fit of a statistical model, which can weigh the complexity of the estimated model and the goodness of the model to fit the data. Bayesian Information Criterions (BIC) can effectively prevent excessive model complexity caused by excessive model accuracy. Models with smaller AIC and BIC have better fit and lesser chance of overfitting (43). Additionally, likelihood ratio tests showed statistical significance (*p* < 0.001). In terms of discriminative power, IDI showed that adding risk factors to the basic model significantly improved discriminative power (*p* < 0.001), which was further confirmed by the ROC and DCA curves (Figures 4, 5).

**TABLE 3:** Prediction accuracy gained by adding risk factors to basic model for the risk of non-suicidal self-injury (NSSI).

	Basic model	Full model
**Calibration**		
AIC	507.90	474.24
BIC	555.67	541.92
LR	Ref.	37.81
LR (P)	Ref.	<0.001
**Discrimination**		
NRI	Ref.	0.005
IDI	Ref.	<0.001
ROC curve	Ref.	2.15
ROC curve P	Ref.	0.143

AIC, Akaike information criterion; BIC, Bayesian information criterion; LR, likelihood ratio; NRI, net reclassification improvement; IDI, integrated discrimination improvement; ROC, Relative operating characteristic curve; Ref., reference group. Basic model included age, marital status, job, education, family history, income, duration of illness, duration of first onset, somatic disease, and BMI.

## 4. Discussion

Using cross-sectional samples combined with modern statistical techniques, we tested the association of demographic information and clinical characteristics with the risk of NSSI among female patients with mood disorders. Using the LASSO regression, we removed other irrelevant variables, and the final results showed that marital status, age of onset, polarity of onset, first-episode psychotic symptoms, social dysfunction, and number of hospitalizations were associated with NSSI. A nomogram model was created to predict NSSI risk in female patients with mood disorders. To our knowledge, this is the first study to implement LASSO regression and nomogram for risk prediction of NSSI in patients with mood disorders.

In our present study, the prevalence of NSSI is 35.6%, which is lower than most of the other studies even though our sample was female inpatients. As mentioned previously, female patients tend to be associated with a higher rates of NSSI (7, 31). Among adolescents, NSSI is often one of the main reasons for receiving psychiatric treatment (44); however, no studies have compared the prevalence of NSSI in adult inpatients versus outpatients with mood disorders. In our research, lower rates of NSSI may be due to the fact that our patients were older and more likely to be married than the participants in other studies, since people who are younger and unmarried are more likely to develop NSSI (22, 25) and adults tend to report lower rates of NSSI due to reattribution or recall bias (45). In addition to the factor above, we also found that patients with fewer hospitalizations were more likely to have self-injury behaviors, indicating that hospitalization can improve the patient’s condition and prevent the recurrence of NSSI. In a day treatment setting for non-suicidal self-injury, NSSI frequency significantly decreased after hospitalization, while quality of life and functional impairment significantly improved at the same time (46). Therefore, our lower incidence of NSSI may also be related to inpatients. The proportion of NSSI varies by research; for example, a study in China found the prevalence of NSSI among inpatients with depression or bipolar disorder to be 62.2% (25), and in another study of Chinese patients with major depressive disorder, they found that 38.6% had committed NSSI in the past year (47). An American study of people with mood disorders found that around 52% of people with bipolar disorder and 37% of people with unipolar depression had at least one episode of NSSI (19). The prevalence of NSSI varied widely among studies due to sample sources and definitions of NSSI. Therefore, more carefully (precisely) designed studies are needed to better understand the epidemiology of NSSI in Chinese patients with mood disorders.

In this study, a stable marital status was one of the protective factors for NSSI in female patients with mood disorders. Stable marital status provides patients with social, financial, and emotional support, and reduces their isolation by providing them with opportunities to interact (48, 49); their spouses’ support in the monitoring of health-related behavior may also help encourage a healthy lifestyle (50, 51). A study was also conducted in Beijing among patients with mood disorders, marital status was compared between patients with and without NSSI and found that being unmarried was a risk factor for NSSI in patients with depression and bipolar disorder (25). There are very few studies on the relationship between NSSI and marital status, most likely due to the fact that research on NSSI mostly involves adolescents who are not yet at the age of marriage. Studies have also found that adolescents with stable parental marital status tend to have less NSSI (52), and marriage has been found as a protective factor for suicide. A meta-analysis of observational studies was conducted to explore the relationships between marital status and suicide, the results indicated that non-married individuals have an aggregate higher suicide risk than married ones (53). The prevention of NSSI through a stable marriage should not be underestimated, though more relevant research is needed to elaborate further in the future.

Meanwhile, some factors that increase the risk of NSSI were mentioned in our study. Early onset of a mood disorder is associated with increased risk of NSSI. Studies have consistently shown that NSSI occurs more frequently in younger age groups, possibly due to difficulty in emotional regulation, adaptive ability, and interpersonal relationships (54–56), and the risk declines with age (22, 57); therefore, early onset of mood disorders may suggest a higher risk of NSSI. Previous article mentioned that age and duration of illness were related to NSSI in patients with mood disorders. By comparing depression patients with and without NSSI, they found increased risks of NSSI in depressed patients related to have a young age, and in patients with bipolar disorder, the incidence of NSSI was significantly lower in patients between 31 and 60 years old group than 18?–30 years patients. Finally, duration of illness for more than 10 years were more likely to have NSSI than those less than 3 years. (25). Our results also supported that patients with NSSI are younger and had shorter duration of illness, but age and duration of illness did not show statistical significance in the LASSO regression, likely because LASSO regression eliminated the multicollinearity and removed the interaction between age, duration of illness and age of onset. In clinical work, more attention should be given to the patients with an early age of onset for prevention.

As for the polarity at onset of mood disorders, previous studies consistently showed that depressive onset patients were associated with a significantly higher suicide risk than manic onset patients (58–61). Similarly, we found that patients with depressive onset had a greater risk of NSSI since patients with depression often feel extreme sadness, low self-esteem, decadence, and pessimism, which breed the idea of NSSI.

We found that first-episode psychotic symptoms is a risk factor for NSSI. The presence of psychotic symptoms at the first episode tends to indicate a greater severity of the mood disorder, and a more severe mood disorder is associated with self-harm (19, 62). It has also been found in many diseases, such as schizophrenia and substance abuse, that patients with psychotic symptoms are more prone to develop NSSI (63).

As reported in literature, patients with social dysfunction have a higher risk of suicide. One study investigated social dysfunction in four different cohorts of patients affected by mood disorders and schizophrenia. Data from 4 independent studies were investigated. Behavioral and affective indicators of social dysfunction were derived and operationalized from scales or questionnaire items related to the interaction with relatives, friends and significant people in patients affected by mood disorders. Consistently across studies, social dysfunction was associated with higher suicide risk (64, 65). Similarly, our results showed that patients with social dysfunction were also associated with NSSI. Lack of social skills appears to be an important risk factor for NSSI in both community and clinical samples. Non-clinical adolescents with NSSI experience more difficulty solving social problems, which interferes with the performance of more adaptive social responses (66). Adolescents with NSSI rated themselves lower on academic intelligence and social skills than adolescents without NSSI (67). These results suggest that social dysfunction may be a risk factor to NSSI. Inconsistent with previous article findings that unemployed patients with mood disorders are more likely to have NSSI (25), we found that patients with NSSI were more engaged in mental labor, but occupation was not included as a risk factor in the nomogram after the LASSO regression.

There are several limitations in this study. First, this was a retrospective study, so we could not choose which variables to collect. Future studies should explore more detailed data and laboratory tests to further identify risk factors for NSSI. Second, this present study recruited patients from a psychiatric hospital in Beijing, which meant that all included patients were hospitalized, and most had better educational opportunities and higher income. Therefore, the generalization of this finding is limited and requires further external validation in future studies. Additionally, the cross-sectional study limited the assumptions of causal relationship, so more well-designed longitudinal studies are needed in the future. Finally, we made prediction models only for female patients since females are more likely to develop NSSI and have more prominent problems (7, 31). In the future, all genders will be further explored. Despite the limitations, this study also has several strengths, including the study of a high-impact behavior, use of a clinical sample, the analysis of data available in the medical record, and an advanced analytic technique is employed.

## 5. Conclusion

Patients with NSSI often avoid seeking help because of the pain and stigma associated with the illness. The identification of specific risk factors can enable the development of recommendations for the prevention of NSSI and the implementation of new interventions. Moreover, recognizing risk factors and developing interventions are not only relevant to self-harm, but also to suicide prevention.

In conclusion, our findings indicate that psychotic symptom at first-episode and social dysfunction increased the risk of NSSI. Stable marital status, later age of onset, manic at onset (compared to depression), and timely hospitalization may reduce the risk of NSSI. This predictive model may help facilitate early identification and prevention strategies and further improve the prognosis of NSSI in women with mood disorders.

## Data availability statement

The original contributions presented in this study are included in the article/supplementary material, further inquiries can be directed to the corresponding author.

## Ethics statement

The study protocol was approved by the Ethics Committee of Beijing Anding Hospital. Written informed consent from the patients/participants or patients/participants legal guardian/next of kin was not required to participate in this study in accordance with the national legislation and the institutional requirements.

## Author contributions

XL, IJ, JZ, and JL carried out the recruitment of patients and performed the statistical analysis. SL and DL wrote the manuscript. SS supervised the entire study. All authors had full access to all study data and analyses, participated in preparing this report, and approved of its final submitted form.
